# Ontogeny of ATP hydrolysis and isoform expression of the Plasma Membrane Ca^2+^-ATPase in mouse brain

**DOI:** 10.1186/1471-2202-10-112

**Published:** 2009-09-07

**Authors:** Daniel Marcos, M Rosario Sepulveda, María Berrocal, Ana M Mata

**Affiliations:** 1 Departamento de Bioquímica y Biología Molecular y Genética, Facultad de Ciencias, Universidad de Extremadura, Avda. de Elvas, s/n, 06071 Badajoz, Spain

## Abstract

**Background:**

Plasma membrane Ca^2+^-ATPases (PMCAs) are high affinity Ca^2+ ^transporters actively involved in intracellular Ca^2+ ^homeostasis. Considering the critical role of Ca^2+ ^signalling in neuronal development and plasticity, we have analyzed PMCA-mediated Ca^2+^-ATPase activity and PMCA-isoform content in membranes from mouse cortex, hippocampus and cerebellum during postnatal development.

**Results:**

PMCA activity was detected from birth, with a faster evolution in cortex than in hippocampus and cerebellum. Western blots revealed the presence of the four isoforms in all regions, with similar increase in their expression patterns as those seen for the activity profile. Immunohistochemistry assays in cortex and hippocampus showed co-expression of all isoforms in the neuropil associated with synapses and in the plasma membrane of pyramidal cells soma, while cerebellum showed a more isoform-specific distribution pattern in Purkinje cells.

**Conclusion:**

These results show an upregulation of PMCA activity and PMCA isoforms expression during brain development in mouse, with specific localizations mainly in cerebellum. Overall, our findings support a close relationship between the ontogeny of PMCA isoforms and specific requirements of Ca^2+ ^during development of different brain areas.

## Background

Nerve cells require highly complex mechanisms of Ca^2+ ^regulation since a precise intracellular Ca^2+ ^concentration is needed for the appropriate development and function of these cells. Plasma membrane Ca^2+^-ATPases (PMCAs) are major components of this regulation. They hydrolyze ATP in order to get the energy to transport Ca^2+ ^from the cytoplasm to the extracellular media across the plasma membrane. The presence of four main isoforms (PMCA1-4) encoded by four genes has been shown widely distributed in most eukaryotic cells [1,2]. Therefore, a strong selective pressure has existed to keep all four isoforms through evolution. Conserved domains among PMCA isoforms correspond to essential transport functions, whereas high diversity domains concern regulatory and functional specialization of each isoform [3-5]. The association between tissue expression and specific cellular functions seems to be the key of this diversity. During neural development, the maintenance of resting cytosolic Ca^2+ ^at nM levels is critical for correct differentiation, dendritic growing and neural maturation [6], and PMCAs are major players in this function. We have reported in a previous study the specific distribution of PMCA isoforms linked to specific cell types and maturation cell stages during prenatal development in chick cerebellum [7]. However, the widespread use of mice as a reference laboratory animal, which presents a mainly postnatal brain development, motivated us to investigate the functional and spatiotemporal expression patterns of these proteins in the mouse brain. These aims contribute to fully understand the Ca^2+ ^metabolism in mouse brain during development. Although the mRNA distribution of PMCA isoforms in developing mouse has been reported by in situ hybridization [8], a study at the protein level as well as at the functional level in different areas of developing mouse brain was still lacking. In this work, we have analyzed the ATPase activity and isoforms expression of PMCA during postnatal neural development in the mouse. These results will contribute to a better understanding of the specific role of these proteins in critical moments of Ca^2+ ^homeostasis during brain development.

## Results

### Functional profile of PMCA in different brain areas during development

**Figure 1:**
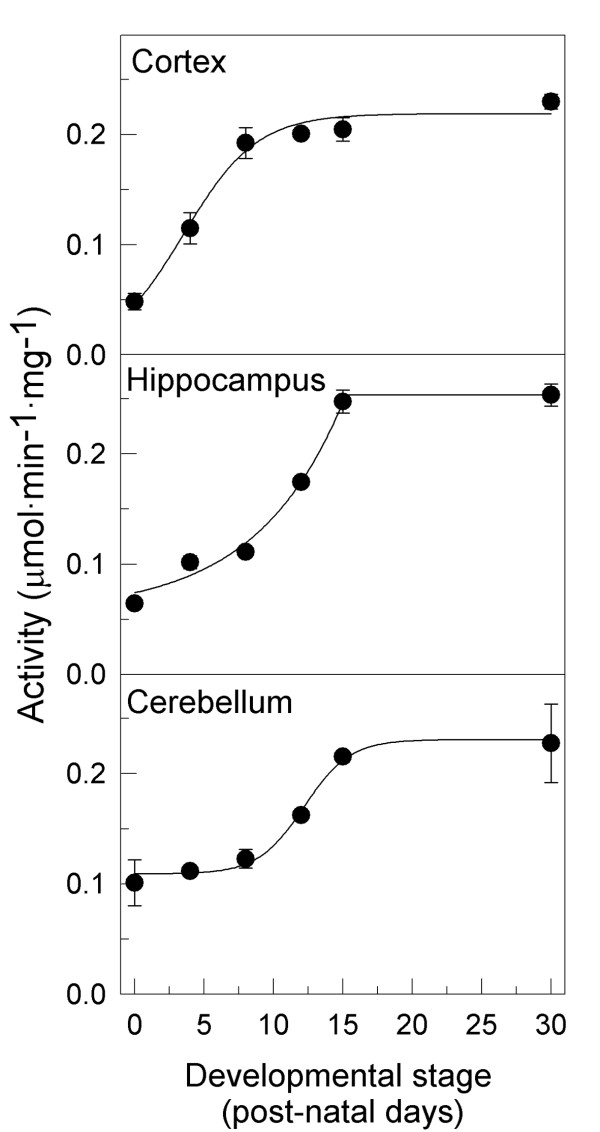
**PMCA activity in developing cortex, hippocampus and cerebellum**. Twenty μg of MV obtained from the indicated brain regions at different developmental stages were used to measure PMCA activity, as described in the Experimental Procedures. Data are the mean ± SE values obtained from three experiments performed in triplicate, and from two preparations.

The PMCA protein showed the capacity to catalyze ATP-hydrolysis in a Ca^2+^-dependent manner from the first stage in all areas (Fig. 1). Besides, the PMCA activity during postnatal development followed two patterns, reaching the *V*_*max *_at P8 in the cortex and at P15 stage in hippocampus and cerebellum. Although the values of *V*_*max *_were similar in all regions (0.237 ± 0.017 μmol·min^-1^·mg^-1^) the increase of activity was more pronounced (around 4-fold) in cortex and hippocampus than in cerebellum (around 2.3-fold).

### Protein expression levels of PMCA isoforms in developing cortex, hippocampus and cerebellum

**Figure 2:**
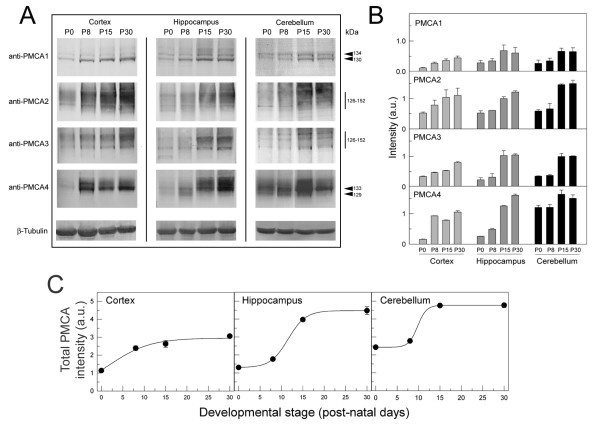
**Immunodetection of PMCA isoforms in MV from different brain regions**. A. MV (20 μg) from cortex, hippocampus and cerebellum at the indicated developmental stages were submitted to electrophoresis, blotted on PVDF membranes and incubated with specific anti-PMCA1, anti-PMCA2, anti-PMCA3 and anti-PMCA4 antibodies. Representative anti-β tubulin immunoreaction as loading control is also shown. B. Quantification of PMCA isoforms (combining all splice variants within each isoform) respect to β tubulin is represented. C. Total PMCA intensity in brain areas obtained from Fig. 2B by adding the band intensities of all isoforms in each stage of development.

The expression of PMCA isoforms during development was examined in the MV by Western blot using isoform-specific antibodies (Fig. 2A). The anti-PMCA1 antibody stained two protein bands whose expression progressively increased with development until P15, in the three analyzed regions. The intensity of the lower band was higher in cortex and hippocampus, while in cerebellum both variants were similarly expressed. These bands seem to correspond to PMCA1a (130 kDa) and PMCA1b (134 kDa) variants, according to Filoteo *et al*. [9]. The PMCA2 antibody stained a spectrum of bands around 126-150 kDa in the three regions, whose intensity progressively increased with developmental stage. This was previously observed in developing chick cerebellum [7]. The anti-PMCA3 antibody recognized three main variants whose expression increased with development. The anti-PMCA4 reaction was very strong in the three regions and increased during development, detecting mainly a protein band that could correspond to variant PMCA4b (133 kDa) and faintly the variant 4a (128 kDa) in hippocampus and cerebellum, according to Filoteo *et al*. [9]. Immunoreaction with β-tubulin (a representative staining from each region is shown) was used as protein loading control for quantification (Fig. 2B). Overall, the largest increase in the expression of total PMCA appeared at P8 stage in cortex and at P15 in the other regions (Fig. 2C), being in good agreement with the kinetic data.

### Isoform-specific localization of PMCA pumps on sagittal sections of developing mouse brain

**Figure 3:**
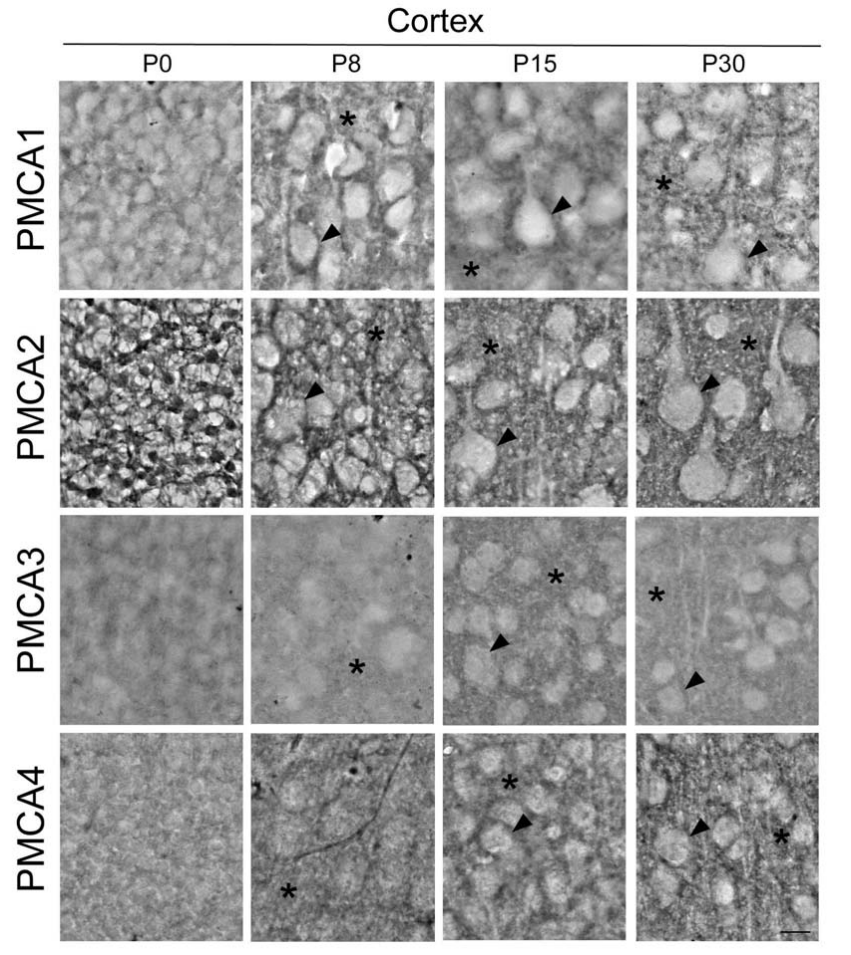
**Localization of PMCA isoforms in parasagittal sections of developing mouse cerebral cortex**. Images show the V layer of the cortex. PMCA1-4 isoforms were detected in developing pyramidal neurons (arrowheads) from the earliest stages to mature cells. PMCA2 was the highest isoform expressed and the PMCA3 the weakest in all analyzed stages. From P8, the four isoforms were located in the neuropil (asterisk) and in the plasma membrane of the bodies of cortical cells (arrowhead). Cytoplasm of the soma and dendritic trunks were immunonegatives. Scale bar: 50 μm.

**Figure 4:**
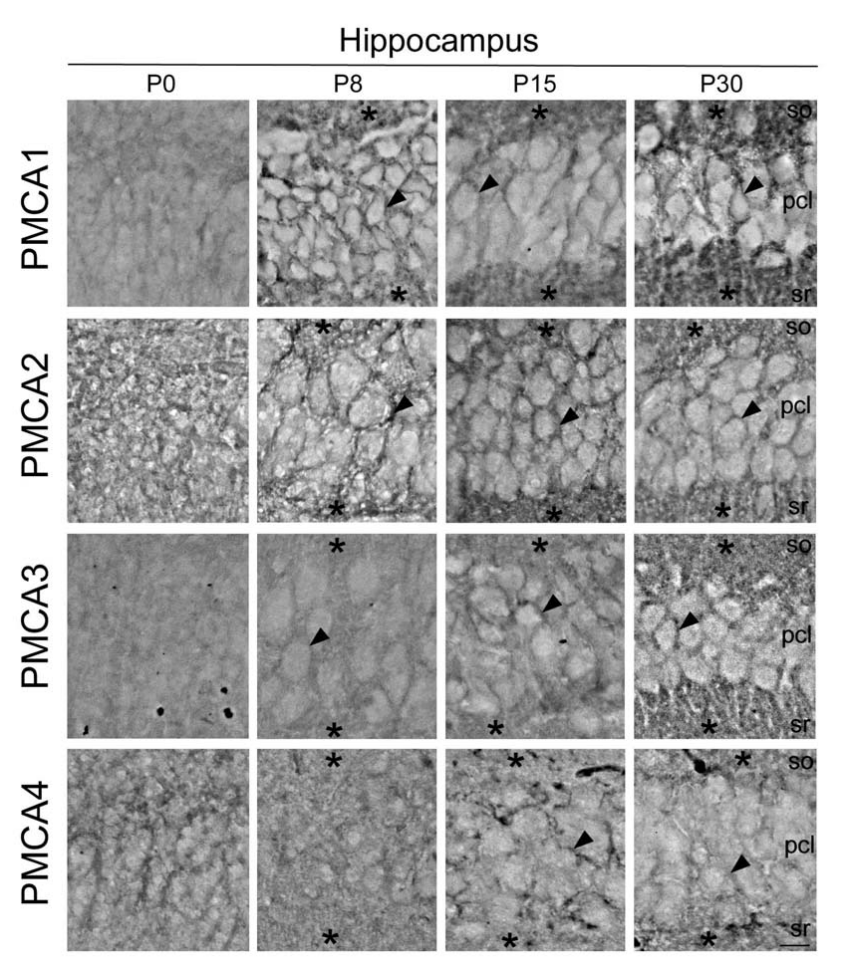
**Developmental distribution of PMCA isoforms in the CA1 area of hippocampal Ammon's horn**. At P0, all isoforms were weakly expressed. Expression levels increase at P8 for PMCA1 and 2 and at P15 for PMCA3 and 4. At P30 stage, all isoforms showed similar immunoreactions. They were located in the plasma membrane of the soma of developing pyramidal neurons (arrowhead) and in the neuropil (asterisk) of *stratum oriens *(so) and *stratum radiatus (sr)*. Cytoplasm of cell bodies was PMCA-negative. pcl, pyramidal cell layer. Scale bar: 50 μm.

**Figure 5:**
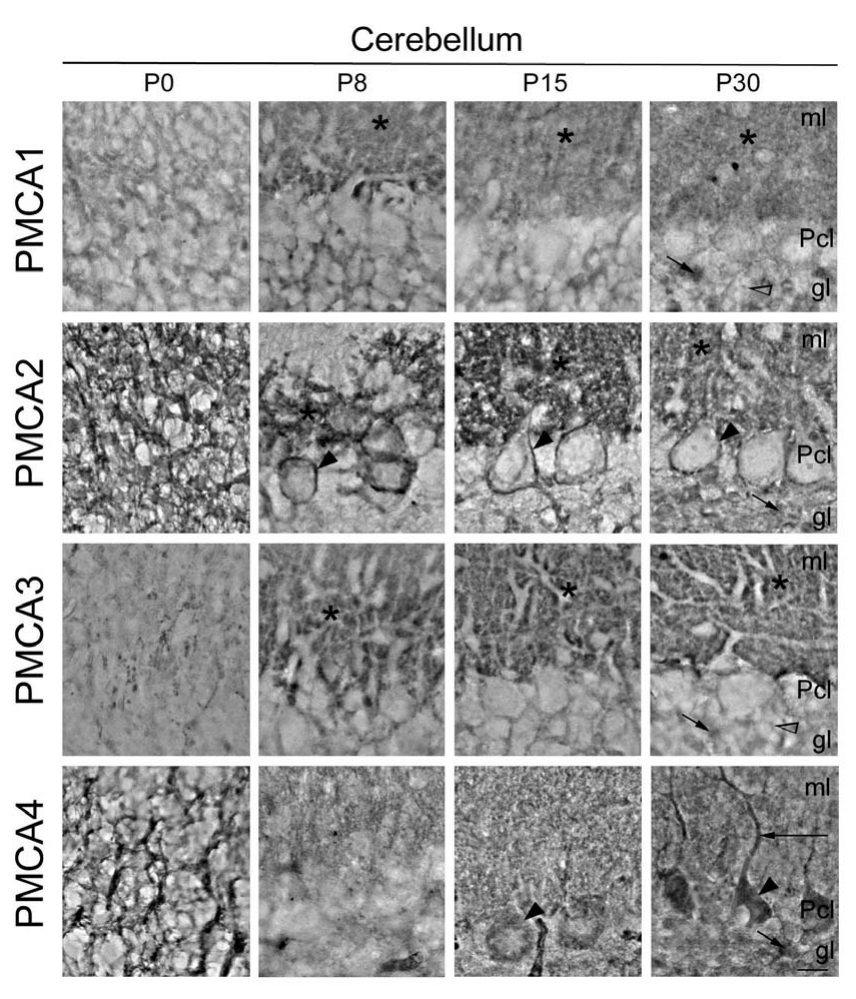
**Localization of PMCA isoforms in developing cerebellar cortex**. At P0, PMCA1 was weakly expressed in cerebellar neural precursors, and from P8 it was located mainly in the neuropil of molecular layer (ml, asterisk). PMCA2 showed high expression levels from P0, being associated to developing Purkinje cells, with a clear localization in the plasma membrane of soma (arrowhead) and dendritic tree (asterisk). PMCA3 showed similar distribution pattern than PMCA1 but with stronger immunoreaction. In contrast, PMCA4 was expressed in the soma of Purkinje cells (arrowheads) from P15, and also in main dendrites at P30 (large arrow). In the granular layer (gl) at P30 stage, while in mature granule cells only were found PMCA1 and 3 (open arrowhead), all isoforms were expressed in cerebellar glomeruli (short arrow). Pcl, Purkinje cell layer. Scale bar: 50 μm.

In order to address if changes in activity and expression levels of PMCAs during development were associated with the maturation of specific cell types, we analyzed their localization in developing brain regions by immunohistochemistry (Figs. 3, 4 &5). The observed distribution was generally consistent with the temporal pattern given by Western blotting. In cerebral cortex (Fig. 3), all PMCA isoforms were expressed in neural precursors at P0, but a more restricted distribution was observed with cell differentiation. The PMCA3 expression was comparatively lower at all stages. At P30 stage, the four isoforms were highly expressed in the neuropil and also in the plasma membrane of the soma of pyramidal cells. The cytoplasm of bodies and dendritic trunks were always PMCA-negative. In the hippocampal Ammon's horn (Fig. 4), the four isoforms were weakly stained in poor-defined structures at the earliest stages. At P8, cellular layers are already established and PMCA1 and 2 were the highest expressed isoforms. They were located in the plasma membrane of the soma of developing pyramidal neurons and showed an intense punctuate staining throughout the neuropil of *stratum oriens *and *radiatus*. At P30, all isoforms appeared with similar expression levels and distribution. In contrast to the other regions, the developing cerebellum revealed clear differences of distribution among PMCA isoforms (Fig. 5). Thus at P0, PMCA1 and 3 showed a lower expression in cerebellar neural precursors than PMCA2 and 4. From P8 on, PMCA1 and 3 were observed as intense punctuate staining in the neuropil within molecular layer. PMCA2 was distributed in similar cellular regions as PMCA1 and 3, being clearly located in the plasma membrane of the soma and dendritic tree of Purkinje cells. By contrast, PMCA4 at P30 stage was mostly detected in the soma and primary dendritic trunk of Purkinje cells. In the granular layer, mature granule cells were PMCA1- and PMCA3-positives, while all isoforms seem to be expressed in cerebellar synaptic glomeruli.

**Figure 6:**
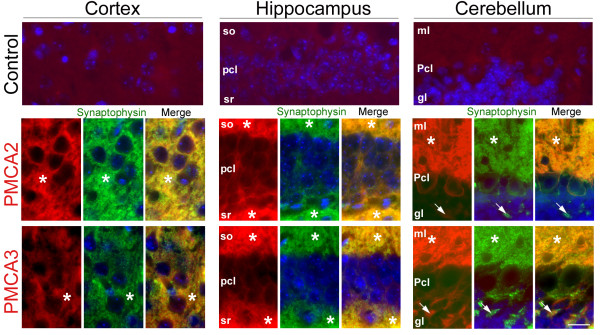
**Double immunofluorescence of PMCA2 or PMCA3, and synaptophysin in different brain regions at P30 stage**. Control slides without the primary antibody are shown to verify specificity of the fluorescence signals. PMCA2 and PMCA3 (red) colocalized with synaptophysin (green) in the neuropil (asterisks) and in cerebellar glomeruli (short arrow), confirming the presence of these pumps in synaptic areas (merge). DAPI staining (blue) was used to visualize nuclei. so, *stratum oriens*; pcl, pyramidal cell layer; sr, *stratum radiatus*; ml; molecular layer, Pcl; Purkinje cell layer, gl; granular layer. Scale bar: 25 μm.

To analyze in detail if PMCA distribution in the neuropil and cerebellar glomeruli corresponds with a synapse distribution, double immunohistochemistry assays were performed in the three regions at P30, using anti-PMCA2 or anti-PMCA3 antibodies (that differ in staining pattern in various areas) as template and the synaptic marker synaptophysin (Fig. 6). A clear and specific co-localization of PMCAs with synaptophysin was observed, thus confirming the presence of these pumps in synaptic areas.

## Discussion

In this work we report the functional presence and immunolocalization of PMCA isoforms during postnatal development of mouse cortex, hippocampus and cerebellum, three major regions implicated in processes of learning and stereotype movements. The functional analysis of PMCA activity revealed that these pumps are active from birth and they are upregulated during brain development. The PMCA activity was comparatively higher than the activities of the two intracellular Ca^2+ ^pumps, SPCA [10] and SERCA (unpublished results) in developing mouse, pointing up the high contribution of PMCAs to the ATP-dependent Ca^2+ ^transport throughout development. This early necessity of Ca^2+ ^regulation in neural precursors is in good agreement with that found in chick cerebellum during prenatal development [11], proving the importance of Ca^2+ ^signaling in early events of neural development. As in chick, PMCA activity profile in mouse was closely related with the time-dependence of isoform expression. We observed in cerebral cortex a fast increase of PMCA activity during the first postnatal week, which is associated with a similar temporal augment of PMCA1-4 isoform expression. However, in hippocampus and cerebellum a slow increase in PMCA activity was observed, also related to a later upregulation of PMCA isoforms expression. These differences reflect special Ca^2+ ^requirements during development of different neural regions.

The increase of PMCA activity is accompanied by changes in isoform-expression and distribution, in correlation with functional cell maturation. In fact, the major increase of PMCA activity mainly occurs during the period of greatest synaptic development. The presence of different PMCAs in the neuropil from early developmental stages and the co-localization with the synaptophysin protein, point us to the contribution of these Ca^2+ ^pumps in the formation and maturity of synapses. This synaptic distribution of PMCAs has been also observed in developing and adult rat [12-14], adult pig [15] and developing chick [7,11], indicating that their localization is conserved among species.

The expression of all PMCA isoforms was quite similar in cortex and hippocampus, but differs from that in cerebellum. Thus, the mouse cerebellar PMCA1 and 3 showed the same distribution pattern as in adult rat [12], but differ from that found in developing chick [7] where they are restricted to the soma and primary dendritic trunk of Purkinje cells. The cerebellar PMCA2 was likewise localized in different species [7,12], while PMCA4 showed similar distribution to that observed for PMCA1 and 3 in chick cerebellum [7]. Furthermore, the localization of PMCA4 in developing mouse hippocampus differs from that reported in developing rat, where it was confined to the soma and dendrites of few scattered cells in CA1 [14]. The mentioned differences could be due to species-dependent expression, or variation between pre- and post-natal neural development. Besides, recent studies in rat cerebellum report a postsynaptic localization for PMCA2 and a presynatic location for PMCA3 [16], which support our results.

The precise targeting of each PMCA isoform (summarized in Table 1) to definite cellular areas and their overlapping localization in others, albeit at diverse expression levels, can be explained by different roles of PMCA isoforms in neural Ca^2+ ^homeostasis. In fact, specific spatio-temporal patterns of PMCA isoforms have been also reported in other excitable tissues with different tasks, as developing retina [17,18], and cochlea [19]. On the other hand, PMCA isoforms differ in their kinetics and affinity for Ca^2+ ^and calmodulin [20], and are also differentially regulated by phosphorylation [20], proteolysis [21,22], or acidic phospholipids [1]. Thus, the expression in a cell type or subcellular region does not necessarily imply functional redundancy, since diverse Ca^2+ ^regulation exists in different compartments of the cell during development. Besides, other extrusion systems expressed in PMCA-positive areas as the Na^+^/Ca^2+ ^exchanger [14], SERCA [11] or SPCA [10], also show upregulation during development. Moreover, each PMCA isoform may be integrated in multiprotein complexes that include PMCA-regulatory proteins, and that are involved in specific and localized Ca^2+ ^signals, e.g.: it has been described that PMCA2 and PMCA4 can bind to Na^+^/H^+ ^exchanger regulatory factor [23] or proteins of the membrane-associated guanylate kinase family [24], respectively. On the other hand, they can be differentially integrated in membrane sub-domains implicated in signal transduction [25,26]. The presence of PMCA isoforms in mouse cortex, hippocampus and cerebellum, from the earliest post-natal stages, and their differences in localization and content may well be associated to isoform-dependent roles in Ca^2+ ^signaling during neural development. This is supported by studies performed in PC12 neural-cell type, where suppression of PMCA2 and 3 expression slowed neurite extension and reduced cell survival [27]. Furthermore, recent observations of PMCA isoform-expression changes in breast cancer cell lines [28] and during cancer cell differentiation [29] also support the importance of PMCAs in cell differentiation.

**Table 1:** Summary of the expression and relative abundance of PMCA1-4 isoforms in main neurons of cortex, hippocampus and cerebellum from developing mouse.

		**Cortex**				**Hippocampus**				**Cerebellum**			
		
		**P0**	**P8**	**P15**	**P30**	**P0**	**P8**	**P15**	**P30**	**P0**	**P8**	**P15**	**P30**
PMCA1	Cell body	(+)	+	+	+	(+)	+	++	++	(+)	(+)	+	+
	Neuropil	(+)	++	++	++	(+)	+	++	+++	(+)	+	++	++

PMCA2	Cell body	(+)	+	++	++	(+)	+	++	++	+	++	+++	+++
	Neuropil	(+)	++	++	+++	(+)	+	++	++	+	++	+++	+++

PMCA3	Cell body	(+)	(+)	+	+	(+)	(+)	+	++	(+)	(+)	+	+
	Neuropil	(+)	+	+	++	(+)	+	++	+++	(+)	++	++	++

PMCA4	Cell body	(+)	(+)	+	+	(+)	(+)	+	+	(+)	(+)	+	++
	Neuropil	+	+	++	++	(+)	+	+	+	+	+	+	+

(+), weak; +, present; ++, strong; +++, very strong.

## Conclusion

The findings reported here show an upregulation of PMCA activity and PMCA isoform expression during brain development, related with a general coexpression of isoforms in cortical and hippocampal regions and different localization in cerebellum. This study provides insights into the specific implications of PMCA isoforms in Ca^2+ ^homeostasis during morphological and functional neuronal maturation.

## Methods

### Materials

Thapsigargin was from Sigma (Madrid, Spain) and ammonium vanadate from Merck (Darmstadt, Germany). The polyclonal anti-PMCA1, anti-PMCA2 and anti-PMCA3 and the monoclonal anti-PMCA4 antibodies, were from Affinity Bioreagents (Golden, CO, USA). The sequences of their epitopes are described in Filoteo et al. [9] for rat and human, and are also conserved in mouse. The anti-β tubulin and anti-synaptophysin monoclonal antibodies were from Sigma. The secondary antibodies conjugated with peroxidase, those biotinylated and the ExtrAvidin-peroxidase were obtained from Sigma, and the fluorescent secondary antibodies labelled with Alexa488 or Alexa594 were from Molecular Probes (Eugene, OR, USA). All other reagents were of the highest purity available.

### Preparation of membrane vesicles

Swiss mice at stages P0, P8, P15 and P30 (postnatal days 0-30) were used to prepare membrane vesicles (MV) from cortex, hippocampus and cerebellum, following the protocol described in Sepúlveda et al. [30]. Animals were manipulated according to the policies on the use of animals in research. Briefly, tissues were homogenized in 10 mM Hepes/KOH pH 7.4, 0.32 M sucrose, 0.5 mM MgSO_4_, 0.1 mM phenylmethylsulfonyl fluoride, 2 mM 2-mercaptoethanol, and protease inhibitor cocktail (Roche Diagnostic, Mannheim, Germany). The homogenate was centrifuged for 10 min at 1500 × g at 4°C. The pellet was discarded and the supernatant was centrifuged for 45 min at 100 000 × g at 4°C. The final pellet was resuspended in 10 mM Hepes/KOH pH 7.4, 0.32 M sucrose and stored at -80°C until use. The protein content was evaluated by the Bradford method [31].

### Electrophoresis and Immunoblotting

Electrophoresis was performed in 6.5% (w/v) Laemmli gels [32]. Proteins were electrotransferred to polyvinylidene difluoride (PVDF) membranes using a Trans-Blot SD semidry system (Bio-Rad). The PVDFs were blocked in a Tris-buffer saline (TBS) containing 2% (w/v) of non-fat dry milk (TBS-milk) for 30 min. After several washes in TBS-Tween 0.05% (v/v), membranes were incubated overnight at 4°C with the polyclonal antibodies anti-PMCA1, anti-PMCA2 and anti-PMCA3 or the monoclonal antibodies anti-PMCA4 (1:1000) and β-tubulin (1:1000). Then, the PVDFs were washed extensively with TBS-milk and incubated for 1 h at room temperature with anti-mouse or anti-rabbit peroxidase-conjugated antibodies (1:3000). The immunoreaction was visualized using 4-methoxy-1-naphtol as substrate.

### Ca^2+^-ATPase activity

The enzymatic activity was measured in MV by using a coupled enzymatic assay at 37°C in specific conditions to measure only the contribution of PMCA activity to total (Ca^2+^+Mg^2+^)-ATPase activity, as described in Sepulveda et al. [30]. Briefly 20 μg of MV were incubated for 4 min with a reaction mixture containing 50 mM Hepes/KOH pH 7.4, 100 mM, KCl, 100 μM CaCl_2_, 2 mM MgCl_2_, 5 mM NaN_3_, 100 μM 1,2-bis(2-minophenoxy)ethane-N, N, N', N'-tetraacetic acid (3.16 μM free Ca^2+^), 0.22 mM NADH, 0.42 mM phosphoenolpyruvate, 10 IU of pyruvate kinase, 28 IU of lactate dehydrogenase and 0.01% saponin (1 mL final volume). The reaction was started with 1 mM ATP and subsequent activity measurements were done after independent additions of 100 nM thapsigargin (to inhibit the presence of SERCA activity), of 2 μM vanadate (to selectively inhibit PMCA activity) and of 3 mM EGTA (to measure Mg^2+^-ATPase activity). The SPCA activity was calculated by subtracting the Mg^2+^-ATPase activity from the activity in the presence of thapsigargin and vanadate. The PMCA activity was calculated then by subtracting the Mg^2+^-ATPase activity and the SPCA activity from the ATPase activity in the presence of thapsigargin.

### Tissue preparation for immunohistochemistry

Mice from stages P0, P8, P15 and P30 were anesthetized with chloroform and subsequently the brains were fixed by transcardiac perfusion with 4% (w/v) paraformaldehyde in phosphate buffered saline solution (PBS) and post fixed by immersion in the same solution for 24 h at 4°C. Brains were rinsed in PBS, cryoprotected in 10% (w/v) sucrose in PBS, and embedded in 10% (w/v) gelatin, 10% (w/v) sucrose in PBS. The blocks were frozen in isopentane cooled at -70°C by dry ice, and stored at -80°C. Serial parasagittal sections of 20 μm were collected on Super-Frost Plus slides using a cryostat Leica CM1900.

### Immunohistochemistry

Sections were permeabilized by immersion in PBS-0.05% (v/v) Triton X-100 (PBS-T) for 15 min, and the endogenous peroxidase activity was quenched with PBS-0.5% (v/v) H_2_O_2 _for 45 min. Sections were blocked in a solution containing 0.2% (w/v) gelatin, 0.25% (v/v) Triton X-100 in PBS (PBS-G-T) and 0.1 M lysine for 1 h. Afterward, sections were incubated in a humidified chamber overnight at room temperature with the anti-PMCA1, anti-PMCA2, anti-PMCA3 or anti-PMCA4 antibodies (1:50). Subsequently sections were washed in PBS-T and incubated with biotinylated goat anti-mouse or anti-rabbit antibodies (1:200) and followed by ExtrAvidin-peroxidase (1:200). The immunodetection was carried out with 0.03% (w/v) 3,3'-diaminobenzidine tetrahydrochloride (DAB). The sections were dehydrated and mounted with Eukitt for their observation under an Olympus CH20 microscope. Alternatively, double immunofluorescence assays were performed with anti-PMCA2 or anti-PMCA3 combined with anti-synaptophysin (1:500), and using the secondary antibodies Alexa594 goat anti-rabbit and Alexa488 goat anti-mouse (1:750). Then, the sections were incubated with 4',6-diamidino-2-phenylindole (DAPI) to stain the nuclei, and covered with FluorSave mounting medium. Results were visualized with an Olympus IX81 fluorescence microscope. Negative controls were performed for every set of experiments by omitting the primary antibody.

### Data analysis

Data were analyzed with SigmaPlot 10.0 software. Quantification of signal intensity was done using Adobe Photoshop 7.0.

## Abbreviations

DAPI: 4',6-diamidino-2-phenylindole; PBS: phosphate buffered saline; PMCA: plasma membrane Ca^2+^-ATPase; PVDF: polyvinylidene difluoride; TBS: Tris buffered saline.

## Authors' contributions

DM performed most experiments and analysed the data. MB co-worked on preparation of some membrane vesicles and preliminary activity assays. MRS and AMM designed the experiments, analysed the data and wrote the manuscript. All authors read and approved the manuscript.
